# CVJA position statement

**Published:** 2013-06

**Authors:** Paul Brink

**Affiliations:** Acting Editor-in-Chief

In the wake of the death of the founder and editor-in-chief of the *Cardiovascular Journal of Africa (CVJA)*, Prof Andries Brink, the Journal continues to operate on the excellent foundation he and managing editor Julia Aalbers established.

The *CVJA* had, at the end of May, an all-time record number of manuscript submissions; already 85% of the total submitted during the course of 2012. We are also delighted to have received submissions from a greater number of African countries and covering an increasing range of fields pertinent to cardiovascular medicine. As a result, we have invited additional topic editors in our established fields and will have to create new fields of interest.

Furthermore, an increasing number of French-language submissions from African countries, accompanied by an English translation of the abstract, have been very welcome. During the 11th PASCAR meeting hosted in Dakar, Senegal, and co-hosted by the Senegalese Society of Cardiology (SOSECAR), great progress was made in sourcing appropriate reviewers for these French-language submissions. This will enable the *CVJA* to further expose the work of our Francophone colleagues globally.

Interviews with selected candidates for the position of editor-in-chief are planned and an announcement will be made soon. In the interim, the *Journal* is being steered by Prof Paul Brink. In this he has relied on advice and support from Profs Amanda Lochner and Johann Brink. Our sincere thanks are extended to them and the topic editors who have tirelessly managed our submissions.

Julia Aalbers, after some uncertainty, has decided to leave the journal to pursue other interests; an intention she had indicated late in 2012. Julia dedicated 25 years to the *CVJA* and was a regular sight at academic gatherings. She is well known for her optimistic can-do attitude and creativity, and has contributed to highlighting Africa on international platforms. We will certainly miss her boundless energy and skill.

From these solid foundations, the CVJA is proudly marching into the future.

